# Ubiquitin-conjugating enzyme membrane anchor Cue1 confers resistance to hygromycin B in *Saccharomyces cerevisiae*

**DOI:** 10.17912/micropub.biology.002032

**Published:** 2026-05-14

**Authors:** James A Avaala, Melissa Palackel, Ian M Tesch, Joseph Gumina, Chance S Creviston, Jason D True, Justin J Crowder, Eric M Rubenstein

**Affiliations:** 1 Department of Biology, Ball State University; 2 The Indiana Academy for Science, Mathematics, and Humanities; 3 Chicago College of Osteopathic Medicine, Midwestern University; 4 Division of Natural, Physical, and Computer Sciences, The Indiana Academy for Science, Mathematics, and Humanities

## Abstract

Aberrant and excess proteins are destroyed by compartment-specific protein quality control mechanisms. In *Saccharomyces cerevisiae* , endoplasmic reticulum (ER)-associated degradation (ERAD) and inner nuclear membrane (INM)-associated degradation (INMAD) require the Ubc6 and Ubc7 ubiquitin-conjugating enzymes. Ubc6 is an integral membrane protein. By contrast, Ubc7 is a soluble protein tethered to the ER and INM membranes by the transmembrane protein Cue1. Here, we assessed the requirement of Cue1 in resisting proteotoxic stress. *CUE1* loss sensitized cells to hygromycin B to a similar extent as *UBC7* deletion, consistent with a shared role for Cue1 and Ubc7 in ER and INM protein quality control.

**Figure 1. CUE1 confers resistance to hygromycin B:** **(A)** By anchoring the ubiquitin-conjugating enzyme Ubc7 to the endoplasmic reticulum and inner nuclear membranes, Cue1 contributes to protein degradation mediated by the Hrd1, Doa10, and Asi ubiquitin ligases. See text for details. **(B-D)** Sixfold serial dilutions of yeast of the indicated genotypes were spotted on medium lacking (No Drug) or containing hygromycin B at the indicated concentrations. Strains in **(D)** were transformed with an empty vector or a plasmid encoding Cue1. Strains used in the experiments in **(B and D)** are derived from the MHY500 genetic background, whereas strains in **(C)** are derived from BY4741. *cue1* Δ #1 and *cue1* Δ #2 are independent clones resulting from homologous recombination-mediated gene replacement. Plates were incubated at 30°C and imaged at the indicated times. Experiments in **(B and C)** were performed three times. The experiment in **(D)** was performed twice.

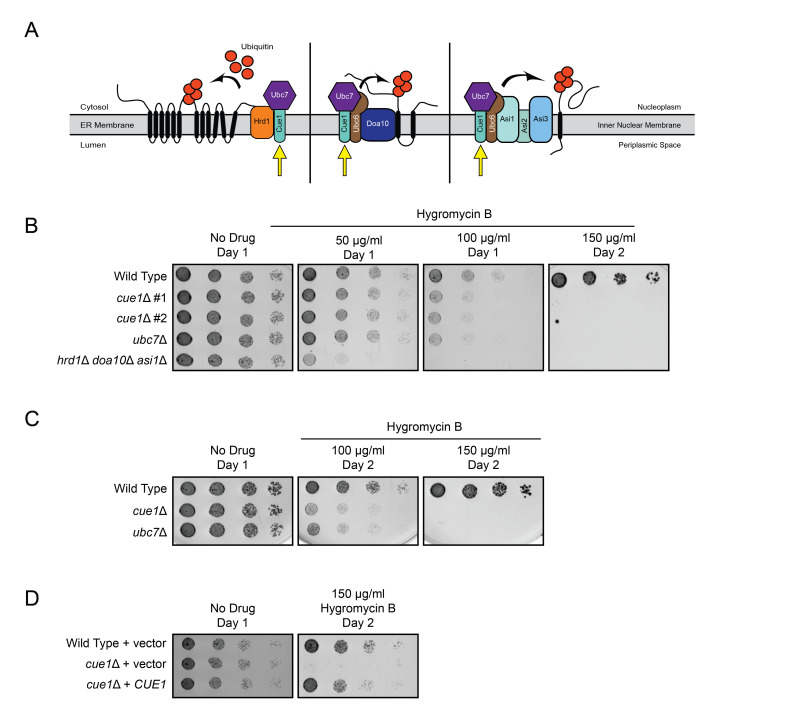



## Description

Cellular and organismal health depends on protein quality control (Badawi et al., 2023; Guerriero & Brodsky, 2012; Higuchi-Sanabria et al., 2018). Aberrant and excess endoplasmic reticulum (ER) and inner nuclear membrane (INM) proteins are destroyed by ER-associated degradation (ERAD) and INM-associated degradation (INMAD), respectively (reviewed in (Mehrtash & Hochstrasser, 2019)). In *Saccharomyces cerevisiae* , the Hrd1 and Doa10 ubiquitin ligases (E3s) mediate ERAD of soluble, transmembrane, and translocon-clogging proteins at the ER membrane (Carvalho et al., 2006; Huyer et al., 2004; Metzger et al., 2008; Rubenstein et al., 2012; Runnebohm, Richards, et al., 2020; Sato et al., 2009; Swanson et al., 2001) ( **Figure 1A** ). Doa10 also localizes to the INM, where it and the heterotrimeric E3 Asi (comprised of Asi1, Asi2, and Asi3) catalyze INMAD of transmembrane INM and soluble nucleoplasmic proteins (Deng & Hochstrasser, 2006; Foresti et al., 2014; Khmelinskii et al., 2014; Swanson et al., 2001). Additional E3s, including Ubr1, Ltn1, and the anaphase-promoting complex, contribute to ER and INM protein quality control (Arakawa et al., 2016; Crowder et al., 2015; Koch et al., 2019; Ruggiano et al., 2016; Stolz et al., 2013).

Ubiquitin-conjugating enzymes (E2s) deliver ubiquitin molecules to E3s for substrate ligation. Hrd1 functions with the E2 Ubc7, while Doa10 and Asi work with two E2s, Ubc6 and Ubc7 (Bays et al., 2001; Foresti et al., 2014; Khmelinskii et al., 2014; Lips et al., 2020; Plemper et al., 1999; Swanson et al., 2001). Ubc7 is anchored at the membrane, stabilized, and activated by the transmembrane protein Cue1 (Biederer et al., 1997; Kostova et al., 2009; Ravid & Hochstrasser, 2007). While a direct Cue1 homolog is not present in mammals, E3s and E3-accessory proteins anchor, stabilize, and activate the Ubc7 homolog UBE2G2 to ensure functional ERAD in mammalian cells (Chen et al., 2006; Das et al., 2009; Smith et al., 2021).

The aminoglycoside hygromycin B distorts ribosome A sites (Brodersen et al., 2000; Ganoza & Kiel, 2001), likely increasing synthesis of the types of aberrant proteins that contribute to age-related neurodegenerative and other diseases (Akbergenov et al., 2025). We and others have demonstrated that several genes required for efficient protein quality control enable cells to resist stress caused by hygromycin B (Akoto et al., 2025; Bengtson & Joazeiro, 2010; Chuang & Madura, 2005; Daraghmi et al., 2023; Flagg et al., 2023; Jaeger et al., 2018; Niekamp et al., 2019; Verma et al., 2013). Deletion of genes encoding ERAD and INMAD E2s and E3s profoundly sensitizes yeast to hygromycin B (Crowder et al., 2015; Doss et al., 2023; Runnebohm, Evans, et al., 2020; Turk et al., 2023; Woodruff et al., 2021). Simultaneous loss of E3s Hrd1, Doa10, and Asi1 causes greater hygromycin B sensitivity than loss of E2s Ubc6 and Ubc7 (Owutey et al., 2024), consistent with compensatory contributions by other E2s to ERAD and INMAD (Bays et al., 2001). Large-scale genetic analyses indicated *CUE1* deletion reduces hygromycin B tolerance (Brown et al., 2006; Dudley et al., 2005), but this result has not been validated in targeted, small-scale studies.

We tested the hypothesis that Cue1 is required for proteotoxic stress resistance. We compared cellular fitness of wild type yeast, two independent *CUE1* knockouts, yeast lacking the E2 Ubc7, and yeast lacking the three major ERAD and INMAD E3s ( *hrd1* Δ *doa10* Δ *asi1* Δ) ( **Figure 1B** ) on media lacking or containing hygromycin B. Deletion of *CUE1* and *UBC7* sensitized yeast to hygromycin B to a similar extent, consistent with a shared role in protein quality control. As previously observed (Owutey et al., 2024), deletion of genes encoding ERAD and INMAD E3s caused a more profound growth defect in the presence of drug. Hygromycin B sensitivity of *cue1* Δ yeast from a distinct genetic background **( Figure 1C )** and *CUE1* plasmid complementation **( Figure 1D )** validate this result.

Hypersensitivity of *cue1* Δ and *ubc7* Δ yeast to proteotoxic stress is consistent with function of a Cue1-Ubc7 subcomplex in ERAD and INMAD (Biederer et al., 1997; Buchanan et al., 2016; Khmelinskii et al., 2014; Pantazopoulou et al., 2016). Future experiments may be conducted to assess whether *cue1* Δ yeast are sensitive to other proteotoxic stressors; however, large-scale studies have demonstrated that *CUE1* promotes resistance to a range of stressors, including transition metals, which oxidatively damage proteins (Ruotolo et al., 2008; Zhao et al., 2020), genotoxic agents (Alamgir et al., 2010; Gaytan et al., 2013; Kapitzky et al., 2010; Ogbede et al., 2021), and sterol biosynthesis disruption (Kapitzky et al., 2010). Our results support a crucial function for Cue1 in maintaining proteostasis in yeast.

## Methods

***CUE1* gene replacement**

To generate yeast strains VJY336 and VJY337, *CUE1* was replaced by *natMX4* through homologous recombination. A 1464-bp *nat4MX4* cassette with termini possessing sequences flanking the *CUE1* gene was PCR-amplified from pAG25 (Goldstein & McCusker, 1999) using primers VJR253 (5’ CGCCATAAAGCATTACAATCTACGATCGCGCAAACTTTTTTCTTTTGGCCCACATACGATTTAGGTGACAC) and VJR254 (5’ TTATGCGCATTATGGGCACACTTGCGTGTTCCCGACAAGCACTTAAGCGTAATACGACTCACTATAGGGAG 3’). The *natMX4* cassette was introduced into VJY6 yeast by lithium acetate transformation (Guthrie & Fink, 2004), followed by selection on medium containing nourseothricin. Successful integration was verified by PCR at 5’ and 3’ recombination junctions.

**Growth assays**

Sixfold serial dilutions of indicated yeast strains were spotted onto yeast extract-peptone-dextrose medium lacking or containing hygromycin B (Gibco) at the indicated concentrations (Guthrie & Fink, 2004) followed by incubation at 30°C for the indicated time, as described in (Watts et al., 2015).

## Reagents

**Yeast strains used in this study.**

**Table:** 

**Name**	**Genotype**	**Figure**	**Reference**
VJY6 (alias MHY500)	*MATa his3-* Δ *200 leu2-3,112 ura3-52 lys2-801 trp1-1 gal2*	1B, 1D	Chen et al., 1993
VJY50 (alias MHY551)	*MATa his3-* Δ *200 leu2-3,112 ura3-52 lys2-801 trp1-1 gal2 ubc7* Δ *::LEU2*	1B	Chen et al., 1993
VJY324	*MATa his3* Δ *1 leu2* Δ *0 met15* Δ *0 ura3* Δ *0 cue1* Δ *::kanMX4*	1C	Tong et al., 2001
VJY336	*MATa his3-* Δ *200 leu2-3,112 ura3-52 lys2-801 trp1-1 gal2 cue1* Δ *::natMX4* Clone 1	1B, 1D	This study
VJY337	*MATa his3-* Δ *200 leu2-3,112 ura3-52 lys2-801 trp1-1 gal2 cue1* Δ *::natMX4* Clone 2	1B	This study
VJY476 (alias BY4741)	*MATa his3* Δ *1 leu2* Δ *0 met15* Δ *0 ura3* Δ *0*	1C	Tong et al., 2001
VJY1075	*MATa his3* Δ *1 leu2* Δ *0 met15* Δ *0 ura3* Δ *0 ubc7* Δ *::kanMX4*	1C	Tong et al., 2001
VJY1098 (MHY11132, ABM297)	*MATa his3-* Δ *200 leu2-3,112 ura3-52 lys2-801 trp1-1 gal2 doa10* Δ *::HIS3 hrd1* Δ *::LEU2 asi1* Δ *::kanMX6*	1B	Mehrtash & Hochstrasser, 2023

**Plasmids used in this study.**

**Table:** 

**Name**	**Description**	**Figure**	**Reference**
pVJ39 (pRS314)	empty vector (CEN, *TRP1* , *AmpR* )	1D	Sikorski & Hieter, 1989
pVJ653 (p414-MET25-Cue1-3xFLAG)	3xFlag-tagged Cue1 driven by *MET25* promoter (CEN, *TRP1* , *AmpR* )	1D	Mehrtash & Hochstrasser, 2022
